# Implementation and Assessment of a Lipoprotein(a) Curriculum for Internal Medicine Residents: A Longitudinal Study

**DOI:** 10.7759/cureus.83859

**Published:** 2025-05-10

**Authors:** Yehuda Eidensohn, Paul O'Rourke

**Affiliations:** 1 Internal Medicine, Johns Hopkins Bayview Medical Center, Baltimore, USA; 2 General Internal Medicine, Johns Hopkins University School of Medicine, Baltimore, USA

**Keywords:** ascvd risk, cardiovascular risk assessment, graduate medical education (gme), lipoprotein(a), medical education curriculum

## Abstract

Introduction: Lipoprotein(a) (Lp(a)) is an independent risk factor for the development of atherosclerotic cardiovascular disease (ASCVD) and is recognized in guidelines as a risk-enhancing factor favoring the more aggressive management of lipids. However, the clinical utilization of Lp(a) testing has been low, especially among internal medicine providers.

Methods: We designed a 30-minute presentation for internal medicine residents that included a general overview of ASCVD risk assessment, differences between Lp(a) and regular low-density lipoprotein cholesterol (LDL-C), and examples of how elevated Lp(a) can change clinical management. Resident confidence in ordering and interpreting Lp(a) was assessed by pre- and post-surveys. Data on Lp(a) ordering practices were abstracted from the electronic medical record for the three months prior to and after the presentation.

Results: The presentation was administered to 22 interns in the fall of the 2024-2025 academic year and was associated with an increase in participant confidence, with mean survey scores increasing from 10 to 24 out of a maximum score of 25 (p<0.001). Lp(a) tests ordered by participants increased from seven (15% of all residents' Lp(a) orders) in the three months prior to 28 (44% of all residents' Lp(a) orders) in the subsequent three months (p=0.008).

Discussion: Lp(a) testing is an underutilized adjunct to ASCVD risk stratification. We developed a brief presentation that was associated with increased confidence regarding the topic of Lp(a) testing as well as changes in practice, as evidenced by increased Lp(a) orders. These encouraging single-center results can help inform future educational efforts on the topic of ASCVD prevention.

Conclusion: An Lp(a) curriculum for internal medicine residents showed promise in addressing the lack of awareness of Lp(a) testing. Further research is needed to assess the generalizability and retention of this curriculum.

## Introduction

Lipoprotein(a) (Lp(a)) is an independent genetic risk factor for the development of atherosclerotic cardiovascular disease (ASCVD) and calcified aortic stenosis [1]. Lp(a) levels greater than 125 nmol/L or 50 mg/dL are associated with at least a 20% increase in risk of ASCVD [2-7]. The American Heart Association/American College of Cardiology 2018 Guidelines on the Management of Blood Cholesterol identified elevated Lp(a) as a risk-enhancing factor favoring the more aggressive management of lipids [8]. Despite the increasing recognition of elevated Lp(a) as an important biomarker in assessing risk for cardiovascular disease, multiple large cohort studies have shown that the clinical utilization of Lp(a) testing has been low [9-11]. 

Our goal was to design and evaluate the impact of an educational intervention on Lp(a) knowledge and ordering practices. We hypothesized that a brief presentation on the topic of Lp(a) testing would increase internal medicine residents' confidence in ordering and interpreting the test and would be associated with increased frequency of testing. 

There are several factors underlying the low uptake of Lp(a) testing. These include the lack of an approved pharmacotherapy, as well as the wide range of recommendations in guidelines regarding who should be tested. European guidelines recommend universal one-time Lp(a) testing [12]. In the United States, guidelines are more heterogeneous. The National Lipid Association recommends testing Lp(a) in all patients with primary severe hypercholesterolemia (low-density lipoprotein cholesterol (LDL-C) ≥190 mg/dL) or premature ASCVD, which the American Association of Clinical Endocrinologists/American College of Endocrinology extends to all patients with established ASCVD [7,13]. The American College of Cardiology/American Heart Association 2018 Guidelines on the Management of Blood Cholesterol do not make a recommendation on screening, stating just that if the result is available, it should be utilized as a risk-enhancing factor [8]. These discordant recommendations may be a source of confusion and can generate uncertainty about both the appropriateness of testing and reimbursement for the test. A reasonable approach may be to order the test when it is expected that the results will change management.

The lack of a targeted treatment for elevated Lp(a) often leads to the question of how it may impact management. Our intervention specifically targeted these areas using case-based learning to illustrate clinical scenarios encountered in everyday practice that are impacted by Lp(a) testing. These scenarios include decisions on initiating or intensifying lipid-lowering therapy when patients have borderline/intermediate ASCVD risk based on the pooled cohort equation; how aggressively to manage comorbid conditions such as obesity, hypertension, and diabetes; and when to refer patients to lipid specialists to discuss alternate therapies and genetic testing. 

Prior studies have shown rates of Lp(a) measurement of 5% or lower even among patients at high risk of ASCVD [11,14]. Rates are especially low among internal medicine providers [14]. Therefore, there is a need for educational interventions targeting internal medicine providers on the topic of Lp(a) with a focus on how elevated Lp(a) can change management. A recent National Lipid Association report called for raising awareness of Lp(a) through education of key stakeholders, including primary care physicians, with a variety of modalities, including live conferences and written material [15]. 

## Materials and methods

We designed a 30-minute presentation that included a general overview of ASCVD risk assessment, differences between Lp(a) and regular LDL-C, and examples of how elevated Lp(a) can change clinical management. Case-based learning was utilized to engage learners in applying Lp(a) to common clinical scenarios. The presentation was incorporated into a series of didactics delivered in the fall of the 2024-2025 academic year to 22 PGY-1 interns at the internal medicine residency program of Johns Hopkins Bayview Medical Center in Baltimore, Maryland, United States. Interns were selected as a convenience sample based on those scheduled to attend the didactics. The presentation slides with links to additional resources were made available to all participants.

A five-question Likert survey assessing participant confidence in ordering and interpreting Lp(a) tests was administered before and after the presentation, using the Qualtrics platform (see Appendices). The survey was created based on an informal review of existing educational surveys commonly used at our institution, and this study served as its validation. Each question was scored between 1 (lowest confidence) and 5 (highest confidence), with a maximum total score of 25. Completing the surveys was optional, and the survey included written informed consent to participate in a research study. Participants were offered a $20 Amazon gift card for completing both surveys. Participant identity was kept confidential by decoupling survey responses from a separate Qualtrics form used for the incentive.

The longitudinal component of the study assessed how this intervention impacted behavior. Data on participants' Lp(a) ordering practices, both inpatient and outpatient, were extracted from the electronic medical record for the three months before and after the presentation. Three months was used to ensure all residents had a similar quantity of clinical time based on their schedules. Orders were attributed to providers by a Core for Clinical Research Data Acquisition specialist who served as an Honest Broker, and only aggregated de-identified data were transferred to the research team. To provide context, we evaluated the entire resident cohort of 64 residents split into four groups: 22 PGY-1 interns who completed the didactic, 10 PGY-1 interns who did not complete the didactic, 16 PGY-2s, and 16 PGY-3s. Demographic information on age, sex, and comorbidities were collected for patients with an Lp(a) test. 

Cronbach's alpha was calculated for the survey with a pre-specified threshold of 0.7 to demonstrate internal validity. Mean confidence scores on pre- and post-surveys were compared with paired t-tests, with a significance level of 0.05. Lp(a) ordering practices and demographics were analyzed using descriptive statistics, with proportion testing using z-tests.

Funding for gift cards and data abstraction was through a grant from the Johns Hopkins Center for Innovative Medicine. The study was approved by the Johns Hopkins University IRB (approval number: IRB00430851). Stata Statistical Software: Release 18 (2023; StataCorp LLC, College Station, Texas, United States) was used for statistical analysis.

## Results

The presentation was administered to a total of 22 interns in the fall of the 2024-2025 academic year. Six of the 22 were preliminary residents who do not have primary care assignments, and the remainder were either categorical internal medicine or primary care interns. 

All 22 participants completed pre- and post-surveys (100% response rate). The survey results demonstrated an increase in participant confidence, with mean (standard deviation) scores increasing from 10(2) to 24(1) (p<0.001). The five-question form demonstrated good internal validity with a Cronbach's alpha of 0.83, above the accepted threshold of 0.7. 

**Figure 1:**
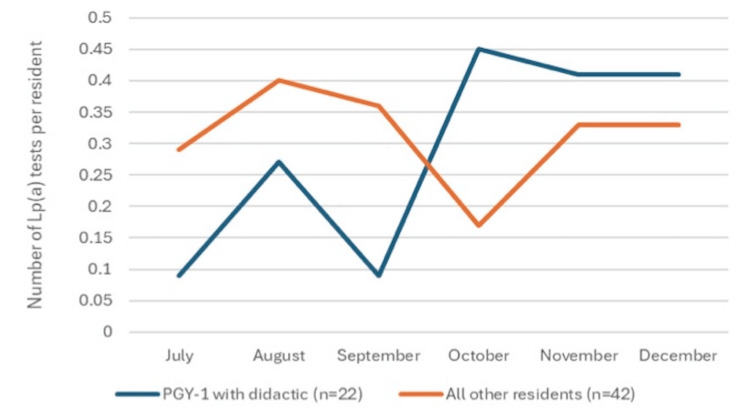
Lp(a) tests per resident Lp(a): lipoprotein(a)

Lp(a) ordering practices were obtained for the entire residency cohort and subdivided into four groups: 22 PGY-1 interns who completed the didactic, 10 PGY-1 interns who did not complete the didactic, 16 PGY-2s, and 16 PGY-3s. Over the preceding three months, all combined PGY groups ordered a total of 48 Lp(a) tests for 47 unique patients. Of all tests, seven (15%) were ordered by participants in the didactic. Twenty-one tests were ordered by PGY-3s, 18 by PGY-2s, and two by PGY-1s who did not participate in the didactic. Testing patterns are shown in Table 1 and Figure 1. The average age of patients with an Lp(a) test was 58 years old; 49% had obesity, 60% had ever smoked, and 45% were female. 

**Table 1:** Lp(a) orders by resident cohort Lp(a): lipoprotein(a)

Resident cohort	July	August	September	October	November	December
PGY-1 with no didactic (n=10)	1	0	1	2	2	1
PGY-1 with didactic (n=22)	2	3	2	10	9	9
PGY-2 (n=16)	8	6	4	2	7	4
PGY-3 (n=16)	3	8	10	3	5	9

In the three months following the presentation, all combined PGY groups ordered a total of 63 Lp(a) tests for 59 unique patients. One patient had three tests, and two had two tests. Of all tests, 28 (44%) were ordered by participants in the didactic, a statistically significant increase compared to the prior three months (p=0.008). Seventeen tests were ordered by PGY-3s, 13 by PGY-2s, and five by PGY-1s who did not participate in the didactic. The average age of patients with an Lp(a) test was 60 years old; 63% had obesity, 37% had ever smoked, and 44% were female. Demographics are shown in Table 2 and were mostly similar between the two time periods.

**Table 2:** Patient demographics

Variable	July-September	October-December	P-value
N	47	59	-
Age, mean (SD), years	58 (17)	60 (14)	0.41
Obesity, N (%)	23 (49)	37 (63)	0.15
Smoking, N (%)	28 (60)	22 (37)	0.02
Female sex, N (%)	21 (45)	26 (44)	0.95

## Discussion

Lp(a) testing is an underutilized adjunct to ASCVD risk stratification, and internal medicine trainees may receive little to no education in this area. We developed a brief presentation that was well received by residents and was associated with increased confidence regarding the topic of Lp(a) testing as well as changes in their practice, as evidenced by increased Lp(a) orders. The magnitude of increase in confidence scores, from 10/25 to 24/25, is likely clinically meaningful, although no minimum clinically important difference was pre-specified. Another strength of the study is the 100% survey response rate, which reduces the risk of bias. This curriculum could be easily incorporated into current ambulatory curricula and may enhance internal medicine residents' confidence in assessing cardiovascular risk in their careers. 

To our knowledge, only two prior studies, both from the University of California, San Diego (UCSD), have focused on interventions to increase Lp(a) testing. Ma et al. demonstrated that an educational program and standardized "checkbox" order sets were associated with an increase in Lp(a) testing among patients undergoing transcatheter aortic valve replacement (TAVR) between 2013 and 2018, culminating in 96% of patients undergoing TAVR in 2018 having been tested for Lp(a) [16]. Bhatia et al. followed up on the results of an additional intervention, modifying the "lipid panel" order set in the entire UCSD Health system to include an option of "lipid panel with Lp(a)" [17]. The total number of patients with an Lp(a) test increased over fivefold from 2010 to 2020, starting with 236 patients in 2010 and peaking at 1458 patients in 2019, declining to 1329 patients in 2020. In their discussion, the authors note that the decline in 2020 may be related to the yearly turnover of trainees and reinforce the need for ongoing educational efforts. 

There is currently no approved pharmacological therapy for elevated Lp(a). Multiple Lp(a)-lowering agents have demonstrated efficacy in lowering Lp(a) and are currently in advanced clinical trials assessing their impact on cardiovascular events. These agents include the antisense oligonucleotide pelacarsen, the small interfering RNAs olpasiran and lepodisiran, and the oral inhibitor muvalaplin [18-21]. As outcomes are reported over the coming years, the landscape of Lp(a) management is likely to undergo significant change, which underscores the importance of current educational efforts to guide the future uptake of therapeutics. 

It is notable that PGY-2 and PGY-3 residents ordered more tests than PGY-1 residents for five of the six months of the study. This is likely attributable to increased clinical exposure to both inpatient cardiology services and outpatient primary care, as well as increased knowledge.

Patient demographics were overall similar between the two time periods. The percentage of smokers reached statistical significance but should be interpreted with caution due to the small sample size and multiple comparisons.

This study has several notable limitations. First, it was performed at a single center with a small sample size and may not be generalizable to other settings. Second, the use of Amazon gift card incentives may have introduced response bias. Third, the study design cannot demonstrate causation, and changes in ordering practices may be due to other causes, including increased clinical exposure to cardiology services. Fourth, the outcome of the Lp(a) test volume did not assess for test appropriateness. The presence of multiple tests for several patients may have been due to provider error, or it is possible that the first orders had not been processed prior to the second order. Additionally, follow-up was limited to three months, and further assessment would be needed to assess for retention and behavior change sustainability.

## Conclusions

Elevated Lp(a) is an established risk factor for ASCVD, and low rates of testing are likely due to a lack of awareness among providers about how Lp(a) testing can influence management. In this single-center study, a brief presentation for internal medicine interns was associated with increased confidence regarding the topic of Lp(a) testing as well as increased Lp(a) orders. Further research is needed to assess the generalizability of this curriculum to senior residents and practicing physicians. Next steps would include six-month and 12-month follow-ups and incorporation into ongoing training.

## Figures and Tables

**Figure 2:**
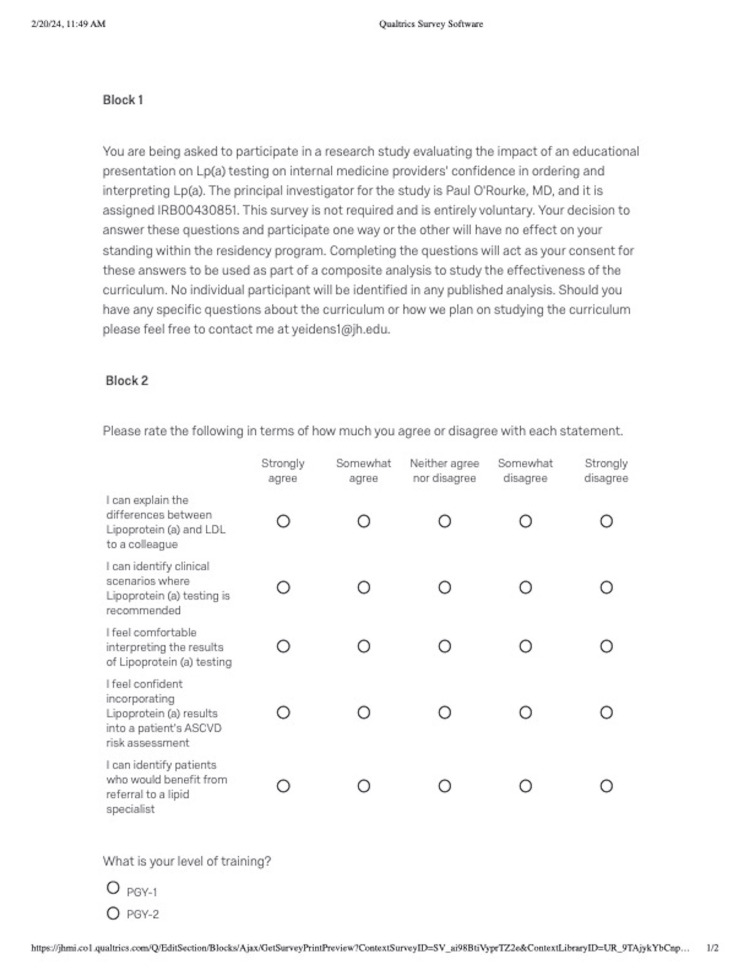
Qualtrics survey
